# A coarse-to-fine restoration framework integrating deep semantic inpainting and texture-aware refinement for Jinling school landscapes

**DOI:** 10.1038/s41598-026-49966-2

**Published:** 2026-04-26

**Authors:** Chuan Yang

**Affiliations:** 1 Sanjiang University, Nanjing, 210012 China; 2 City University of Macau, Macau, 999078 China

**Keywords:** Virtual restoration, Jinling School, Coarse-to-fine framework, Texture grafting, Jimofa, Minimal intervention, Semantic inpainting, Engineering, Materials science

## Abstract

Traditional Chinese ink paintings on paper or silk are highly susceptible to degradation. Over time, physical decay such as creases not only damages the surface but also obscures the original brushwork. Virtual restoration, as a non-contact digital intervention, has emerged as a vital tool for heritage preservation. Yet, generic generative models—most notably GANs and Diffusion—often struggle with the dense, layered textures of the Jinling School(金陵畫派), particularly the *Jimofa* (積墨法) technique. GANs tend to “hallucinate” details that clash with traditional brushwork logic, while Latent-based models can drift toward a modern aesthetic that feels disconnected from the original archaic spirit. To address these discrepancies, we propose a coarse-to-fine framework specifically calibrated for Jinling School landscapes. This coarse-to-fine architecture mirrors the traditional ‘Bone-first, Ink-second’ painting methodology of the Jinling School. By decoupling structural recovery (Skeleton) from texture deposition (Flesh), our computational process aligns physically with the artifact’s original creation logic. Initially, a deep convolutional network restores macroscopic structural continuity, effectively smoothing creases and reclaiming the mountain’s geometric silhouette. This is followed by a texture-aware refinement module that uses manifold texture grafting to inject high-frequency details into otherwise blurred regions. Experimental results indicate that, beyond restoring overall continuity, the framework appears able to recover aspects of the high-frequency “ink noise” and deep tonal peaks traditionally associated with the *Jimofa* technique. Crucially, comparative analysis confirms that the framework significantly reduces the risk of ‘semantic hallucination’ (e.g., the fabrication of non-existent objects) prevalent in large-scale generative models, ensuring distinct historical fidelity. Quantitative assessments—specifically average gradient and edge density—show a measurable improvement over baseline models, all while the system maintains a strict adherence to the principle of “minimal intervention” in undamaged areas. By mitigating the over-smoothing typical of conventional deep learning, this work suggests a path for the digital restoration of rare, small-sample artworks that seeks to balance visual plausibility with historical rigor.

## Introduction

Within the climate-controlled repositories of modern museums, the physical substance of traditional Chinese ink paintings is undergoing a slow, relentless, and irreversible decay. For the surviving masterpieces of the Jinling School—a group of 17th-century artists centered in Nanjing whose rugged northern-style peaks and meticulous Song-dynasty textures stood in stark contrast to the then-dominant Southern School—creases, mold, and material loss are more than mere physical pathologies. They function as visual noise, blocking contemporary audiences, especially the “digital natives” of the younger generation, from grasping the essence of traditional aesthetics^1^.

Digital preservation has become a cornerstone of China’s cultural strategy, with landmark projects such as “Digital Dunhuang” and the “Digital Palace Museum” successfully bringing sequestered archives into the realm of public education. Yet, a “Transmission Paradox” remains. While high-resolution scanning preserves an artwork’s current state, it also dutifully records its degradation. When these degraded images circulate across mobile networks, the fractured lines and severed mountain ridges can mislead the public. For students lacking professional connoisseurship, the legendary *Jimofa*^2^—the technique of accumulating ink layers—loses its rhythmic depth, and the *Cang-run* (蒼潤) atmosphere of archaic moisture vanishes^3^. In this context, virtual restoration is less a technical exercise than an act of “readability enhancement.” It seeks to provide the public with a digital surrogate that, while perhaps lacking absolute historical authenticity, possesses a necessary Aesthetic Integrity.

The field of virtual restoration has witnessed a paradigm shift, transitioning from classical digital image processing to the sophisticated deep generative architectures of today. Early digital restoration efforts relied heavily on diffusion-based and patch-based paradigms. The BSCB model, introduced by Bertalmio et al. (2000), utilized partial differential equations to propagate information from the boundaries of a damaged region inward—a technique that remains notably effective for minor scratches^4^. Yet, these methods often falter when faced with large-scale texture loss. While Criminisi et al. (2004) introduced texture synthesis to fill gaps using exemplar patches^5^, this logic excels in repetitive environments but struggles with the stochasticity of *Qi-yun* (氣韻) inherent in Chinese ink painting^1^. Nevertheless, the philosophy of directly utilizing original pixels for physical filling offers a distinct advantage in maintaining the granular integrity of *Jimofa*^1^, providing the essential spark for the second stage of our proposed framework.

The emergence of Convolutional Neural Networks (CNNs) fundamentally reconfigured the field. Pathak et al. (2016) introduced Context Encoders, bringing adversarial training to image completion^6^, followed by Iizuka et al. (2017), who utilized global and local discriminators to ensure consistency^7^. More recently, Generative Adversarial Networks (GANs) have become the dominant force^8^. Innovations such as the edge-guided diffusion patch GAN^9^ and structure-focused networks^10^ have demonstrated success in mural and painting restoration. Still, GANs are plagued by inherent instability, often leading to “hallucinated textures.” Even as cutting-edge research attempts to merge Diffusion models with GANs^11^, constraining these powerful engines to follow the strict brushwork statistics of a specific school remains a critical gap.

Within the specific domain of cultural heritage digitization, preservation has matured from mere “recording” to active “reconstruction.” Research from teams like Zhejiang University has established high-fidelity standards for microscopic texture retention^12^, while others have utilized computer vision to extract specific “disease layers”^13^. Recent efforts have also turned toward school-specific styles, proposing hierarchical models^14^ and style-aware representation learning^15,16^. Our study draws upon this collective experience while maintaining a firm commitment to the Principle of Minimal Intervention. By utilizing a controlled deep learning model, we aim to safeguard the singular style of the Jinling School while circumventing the ethical hazards of generative over-production.

To navigate this double bind of over-smoothing and generative hallucination, we propose a specialized virtual restoration framework built on a Coarse-to-Fine strategy. We move away from the “black-box” paradigm of end-to-end generation in favor of a dual-phase system. The first phase employs a deep convolutional network to reclaim macroscopic structures and geometric silhouettes. This is followed by a Texture-Aware Refinement module, which introduces physical-level grafting. Rather than chasing a generic restoration capability, we focus strictly on the Nanjing regional landscape—a cultural hub where Ming-dynasty masters observed real scenery to create “habitable” mountainscapes distinct from traditional reclusive styles^2,3^. By curating a specialized dataset of 72 high-resolution masterpieces, we train the model not only to “see” the flow of the mountain but to accurately reconstruct the high-frequency statistical traits of the *Jimofa* technique. Experimental results suggest that this approach significantly enhances image sharpness while strictly adhering to the Principle of Minimal Intervention, ultimately achieving a balance between technical precision and aesthetic fidelity.

## Materials and methods

To resolve the dual crisis of over-smoothing and stylistic distortion in Jinling School restoration, we propose a coarse-to-fine cascaded framework. Following the edge-guidance logic emphasized by Sun et al. (2024)^10^, we decouple the restoration task into two specific sub-problems: macroscopic semantic completion and microscopic texture refinement. This separation is essential. It guides the model to bridge the semantic gap between generic image features and the nuanced, school-specific ink textures that define the medium.

### Dataset construction: from general to domain-specific

**Fig. 1:**
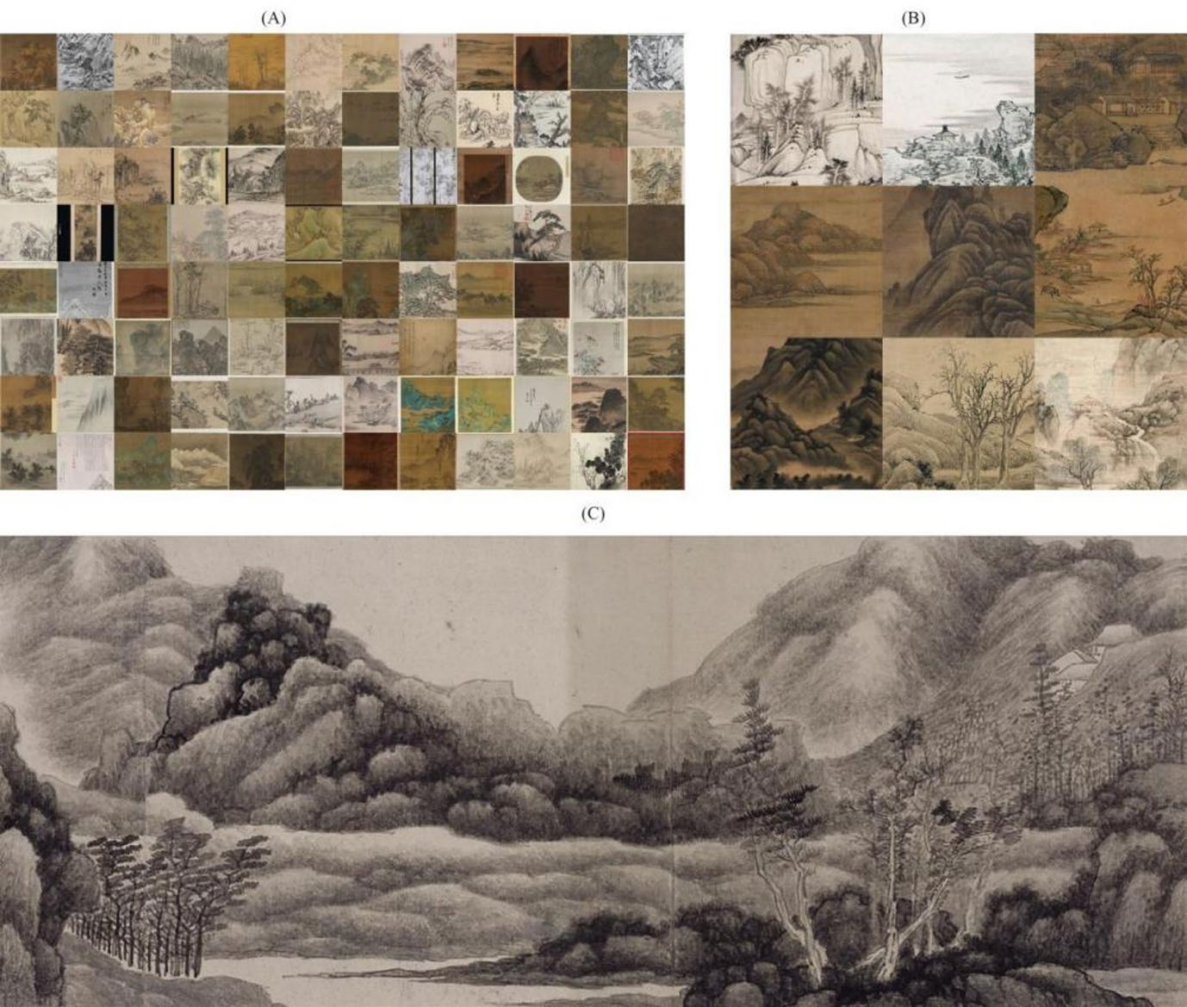
Hierarchical Dataset Construction and Target Artifact. (Top Left) General Landscape Pre-training Set for Stage 1 structural alignment. (Top Right) Jinling School Fine-tuning Set curated for *Jimofa* textures. (Bottom) Target Artifact: *Landscape Scroll* by Gong Xian (Qing Dynasty). This masterpiece, with its dense “Black Gong” style, is the primary subject for validating our framework.

The generalization of any deep convolutional network is limited by its training distribution. Faced with the “small sample problem” of surviving Jinling masterpieces, direct training risks immediate overfitting. We therefore designed a dual-layer dataset strategy (see Fig. 1).

1. The first layer is a General Landscape Pre-training Set featuring ~ 2,000 traditional ink wash paintings. Images were standardized to a short-edge of 1024 pixels and randomly cropped into 15,000 patches (512 × 512). This stage is not about mimicry. As Xu et al. (2023)^14^ noted, the goal is to provide enough topographic diversity to force the model into learning the underlying “compositional grammar” of the genre. To verify this, we ran cross-domain inference on real-world natural scenery. The results were telling. Even with unseen natural data, the model reconstructed perspective and contours consistent with physical reality. It had acquired a robust structural prior.
2. The second layer—the Jinling-Specific Fine-tuning Set—is the core of our method. We curated 72 high-resolution digital reproductions from masters like Gong Xian and Fan Qi. At resolutions exceeding 600 dpi, these files capture the micro-morphology of paper fibers and ink particles^12^. To ensure rigorous evaluation and prevent data leakage, we implemented a strict slide-level split strategy. We randomly selected 60 complete paintings for the training set and reserved 12 distinct paintings exclusively for the test set. This ensures that the patches used for evaluation act as unseen data and never appeared during the training phase.

Preparation for the Stage 2 refinement involved two specific steps:

1. Interference Removal: Colophons and seals were manually excluded via center-cropping.
2. High-Frequency Sampling: We implemented a sliding window (512 × 512, 256-pixel stride). This high-overlap strategy expanded the 72 originals into ~ 3,500 style-intensive patches, effectively amplifying the high-frequency traits of the *Jimofa* technique within our feature library.

Preliminary tests indicated that while Stage 1 effectively repairs structural breaks, it leaves a residue of blurring or “ghosting” in dense ink regions. A single network simply cannot satisfy both structural integrity and textural sharpness under small-sample constraints. Based on this finding, we defined the first stage as “structure-centric,” delegating the resolution of textural artifacts to the physical-level refinement of Stage 2.

### The coarse-to-fine restoration framework

**Fig. 2:**
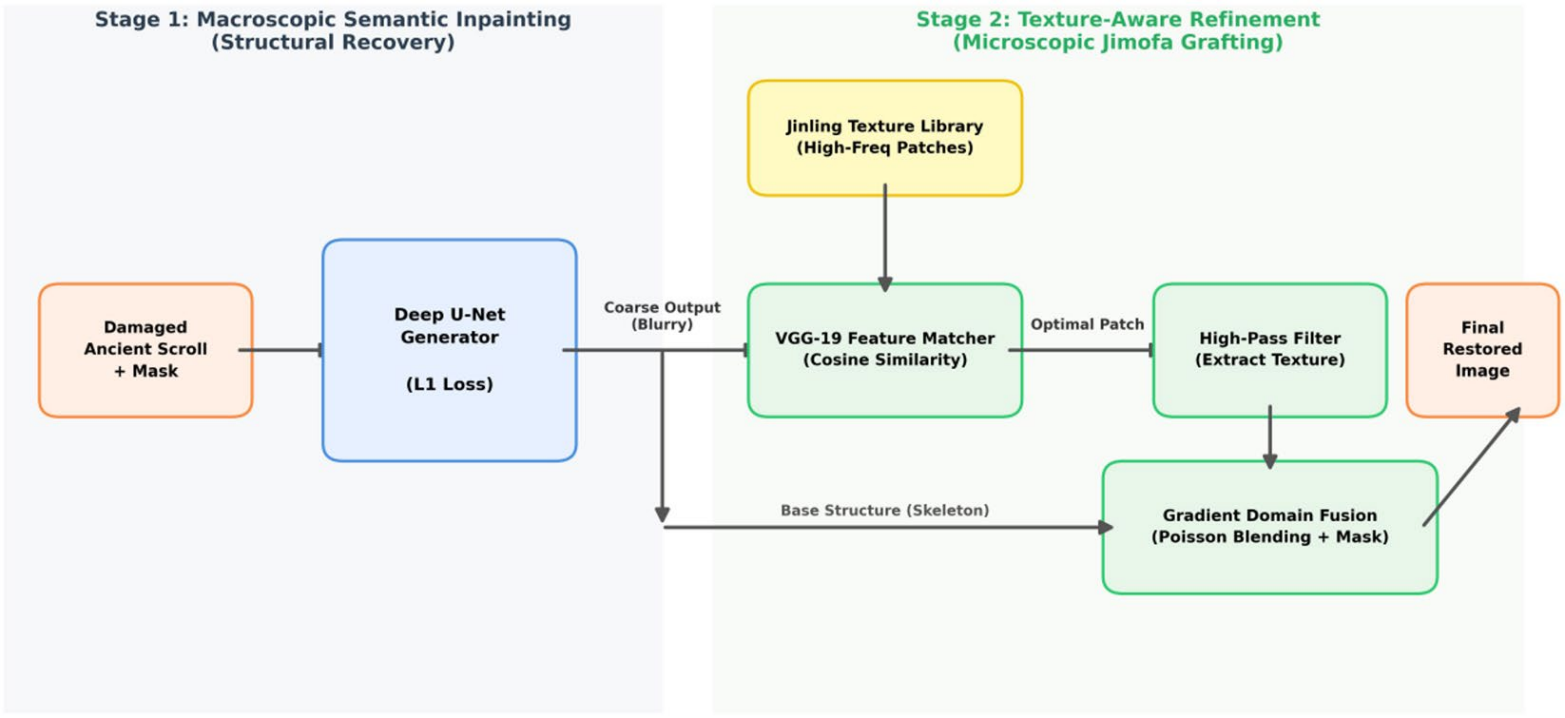
System architecture diagram of the coarse-to-fine restoration framework.

As illustrated in Fig. 2, the overall system architecture visually demonstrates this decoupled philosophy. The pipeline takes a damaged painting as input and first routes it through a macroscopic U-Net to establish a continuous geometric foundation. Subsequently, the intermediate blurry output is processed by the non-parametric VGG-19 feature matcher, which dynamically queries the high-resolution Jinling dataset to graft authentic *Jimofa* textures onto the repaired region.

Balancing structural logic with textural clarity requires a departure from end-to-end “black-box” generation. Our cascaded architecture reflects this shift.

1. Stage 1: Deep Semantic Inpainting. We utilize a classic U-Net^17^ grounded in the Principle of Minimal Intervention^18^. While GANs excel at synthesis, their adversarial nature often triggers “hallucinations”^19^. U-Net’s skip connections, however, allow high-frequency spatial information to bypass compression layers. This ensures the model “infers” missing regions from surrounding semantics rather than re-painting them. The output, $\:{I}_{coarse}$, resolves global geometry but inevitably retains some smoothing—a byproduct of the L1 loss function.
2. Stage 2: Texture-Aware Refinement. This represents the pivotal innovation of our work. To reclaim the granular *Jimofa* texture, as will be demonstrated in the Results section, we designed a non-parametric refinement module. Rather than relying on simple pixel-wise matching, the module employs a perceptual metric to ensure semantic consistency. Specifically, we calculate the cosine similarity between VGG-19 feature maps (extracted at layer relu4_1) of the coarse output ($\:{I}_{coarse}$) and candidate patches in our texture library to identify the optimal $\:{I}_{retrieve}$. These high-frequency components are then fused logically as follows:

1 $$\:{I}_{final}={I}_{coarse}+\alpha\:\cdot\:(M\odot\:\mathcal{H}({I}_{retrieve}\left)\right)$$

**Fig. 3:**
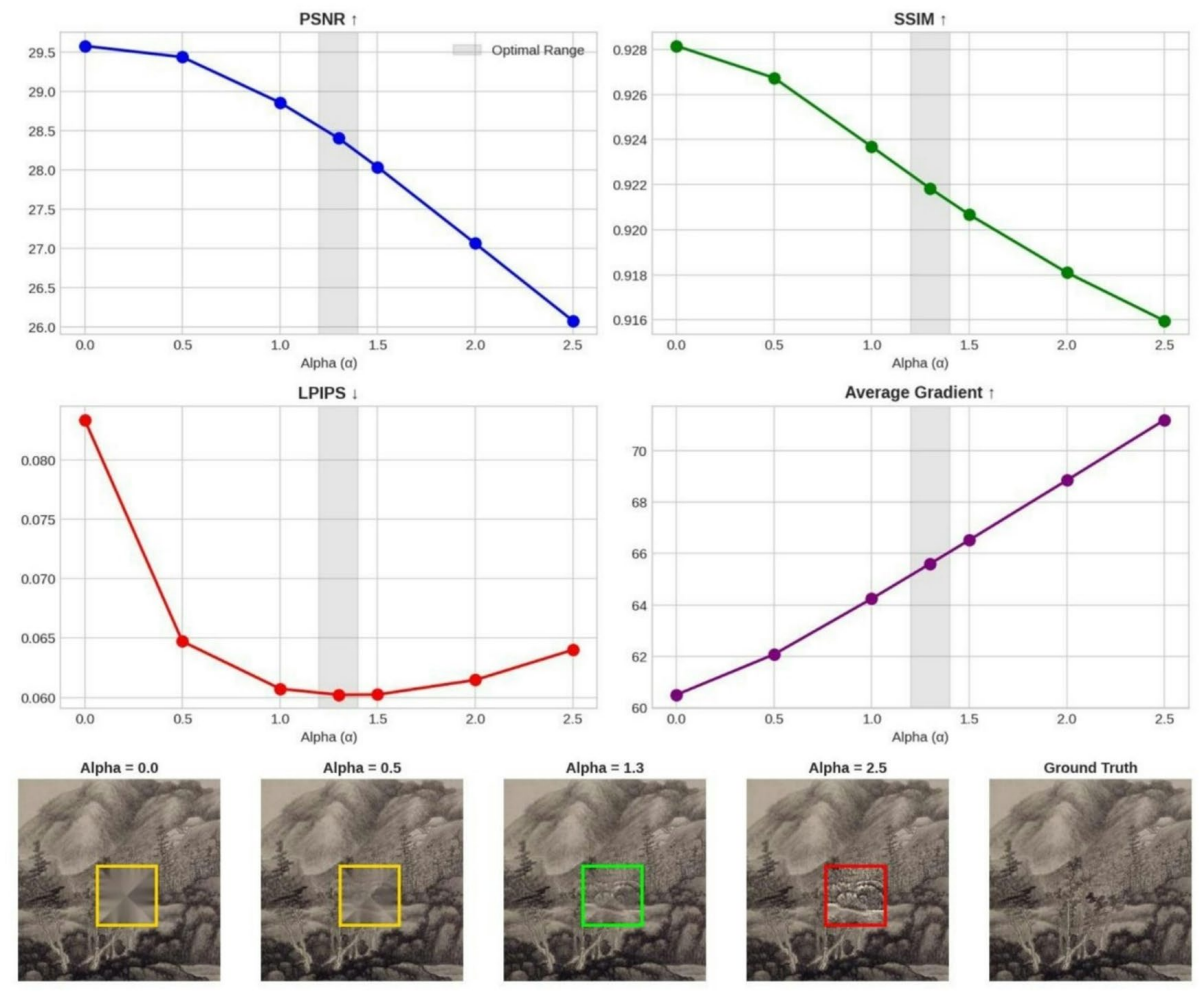
Parameter sensitivity analysis of the enhancement coefficient $\:\alpha\:$.

Parameter Sensitivity Analysis: The enhancement coefficient $\:\alpha\:$ in Eq. (1) is a critical hyperparameter governing texture injection. To justify its selection and demonstrate methodological robustness, we conducted a sensitivity analysis sweeping $\:\alpha\:\in\:\left[\mathrm{0.0,2.5}\right]$. Quantitative trajectory (measuring PSNR, SSIM, LPIPS, and Average Gradient) reveals a classic Perception-Distortion tradeoff. As $\:\alpha\:$ increases from $\:0.0\:to\:1.3$, the Average Gradient steadily improves, capturing high-frequency Jimofa features, while LPIPS drops to its optimal minimum. However, when $\:\alpha\:$ exceeds $\:1.5$ and approaches $\:2.5$, PSNR and SSIM exhibit a sharp decline, manifesting visually as unnatural, high-contrast noise artifacts. Thus, the interval $\:\alpha\:\in\:\left[\mathrm{1.3,1.5}\right]$ serves as the optimal threshold, maximizing texture sharpness without compromising macroscopic structural fidelity. (Fig. 3)

Where $\:\mathcal{H}(\cdot\:)$ denotes a high-pass filter extracting texture details from the retrieved patches, and $\:\alpha\:$ is the enhancement coefficient. Crucially, the binary mask $\:M$ is applied via the Hadamard product ($\:\odot\:$) to confine the texture injection strictly to the damaged areas. However, direct superposition can introduce visible seams at the mask boundaries. To address this, the final pixel integration is implemented using Poisson Blending (Gradient Domain Fusion), which enforces gradient continuity along the boundaries while preserving the Principle of Minimal Intervention.

### Training protocol and optimization

Training focused on the Stage 1 U-Net, as Stage 2 operates on rule-based inference. We followed a strict two-stage protocol:

General Pre-training: 50 epochs on the general dataset to establish basic completion capabilities.

Domain-Specific Fine-tuning: additional 150 epochs (Total: 200 epochs) on the Jinling dataset using pre-trained weights as a baseline.

We prioritized the L1 loss (Mean Absolute Error) for structural consistency. While MSE is common, L1 produces fewer blurring artifacts at edges^13^. The objective is defined as:

2 $$\:{\mathcal{L}}_{rec}=\left|\right|M\odot\:\left(G\right({I}_{masked})-{I}_{gt})|{|}_{1}$$

Here, $\:{I}_{masked}$ represents the corrupted input image, defined as $\:{I}_{gt}$ ⊙($\:1-M$), where $\:Igt$ is the ground truth original image and $\:M$is the binary mask indicating the damaged regions. Unlike generative models based on latent noise z, our generator G takes the masked image directly as input to infer missing semantics. The term $\:M$⊙… ensures the L1 loss is calculated exclusively within the missing regions, preventing the model from altering valid pixel information.

## Results

The validation of our model encompasses both quantitative metrics and multi-dimensional qualitative assessments of visual legibility and stylistic fidelity. Following the hierarchical transfer learning strategy previously described—fine-tuned on 72 original masterpieces—we designed three sets of experiments on the test set. These span from microscopic brushwork to macroscopic structure. They aim to answer three fundamental questions: Can the model repair the subtle physical pathologies that mar the surface? Can it infer plausible mountain structures when information is severely lacking? And, crucially, can it preserve the hallmark *Jimofa* texture during pixel reconstruction?

### Qualitative evaluation: from strokes to structure

We begin with the most pervasive physical pathology in ancient paintings: creases. While ostensibly a simple task of geometric filling, crease restoration demands a profound understanding of “Brush Flow” (筆勢). Creases typically sever the continuity of *Cun-fa* (皴法); conventional filling methods often lead to misaligned or fractured lines.

**Fig. 4:**
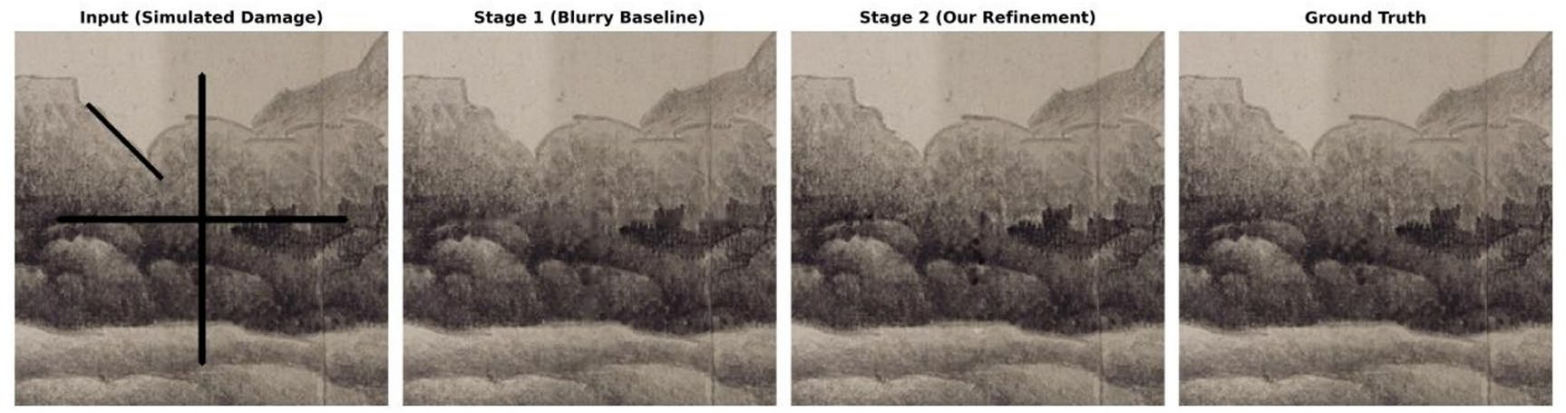
Fine crease restoration results. The proposed method (Ours) effectively restores the continuity of the brush flow across the damaged crease region, preserving the high-frequency “Moss Dot” textures better than baseline methods.

As illustrated in Fig. 4, we tested the model on localized regions containing intricate brushwork. The results demonstrate a capacity for “semantic understanding” that transcends mere pixel-level substitution. In the ridge sample on the left, the model does more than fill the white crease; it successfully reconnects the severed “Moss Dots” (苔點) on either side^10^. Furthermore, the generated ink density integrates seamlessly with the surrounding environment, avoiding any detectable “patchwork” effect. This preservation of high-frequency detail suggests that the model effectively internalized the continuity logic of Jinling School brushwork during the fine-tuning phase.

If crease repair tests the ability to “reconnect,” then large-scale restoration tests the ability to “build mountains.” When faced with irregular voids, the model must “hallucinate” rock structures based on the surrounding context.

**Fig. 5:**
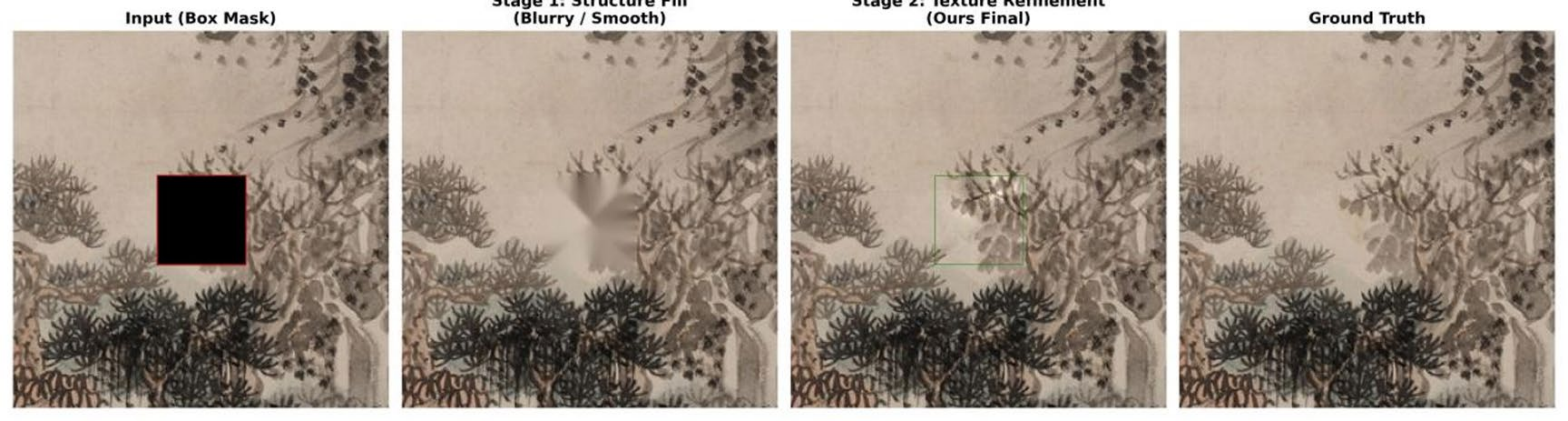
Large-scale structure completion. In scenarios with significant information loss, the model infers plausible rock structures consistent with the surrounding Jinling landscape features, despite a slight smoothing effect in dense ink areas.

Figure 5 demonstrates this in a typical heavy hills and valleys (重丘壑) scene. Rather than simply duplicating adjacent textures, the model generates plausible extensions of rock textures based on the mountain’s flow. We acknowledge that in some extremely dense ink regions, the generated texture appears slightly smoother than the original—a known limitation of pixel-wise objective functions, which inevitably leads to the over-smoothing of high-frequency textures^13^. However, from a structural perspective, the reconstructed contours and Cun-fa (皴法) trajectories remain visually plausible. For digital display and public education, this structural correctness is often more vital than absolute pixel-level precision.

### Quantitative evaluation and style verification

Ablation Study and Natural Image Quality Evaluation: To explicitly isolate the contribution of the texture-aware refinement module, we conducted a quantitative ablation study comparing “Stage 1 only” versus “Stage 1 + Stage 2”. Crucially, to ensure a rigorous evaluation and verify that the model did not merely memorize training data, this experiment was performed on a distinct, unseen landscape painting by Gong Xian, strictly adhering to our slide-level test set separation. As shown in Table 1, relying solely on Stage 1 yields a baseline PSNR of 26.3023 and an Average Gradient of 45.8563, reflecting an over-smoothed output typical of standard semantic inpainting. The integration of Stage 2 significantly improves all metrics, particularly dropping the perceptual error (LPIPS) from 0.0729 to 0.0228, and raising the Average Gradient to 50.6889, explicitly validating the necessity of manifold texture grafting.

**Fig. 6:**
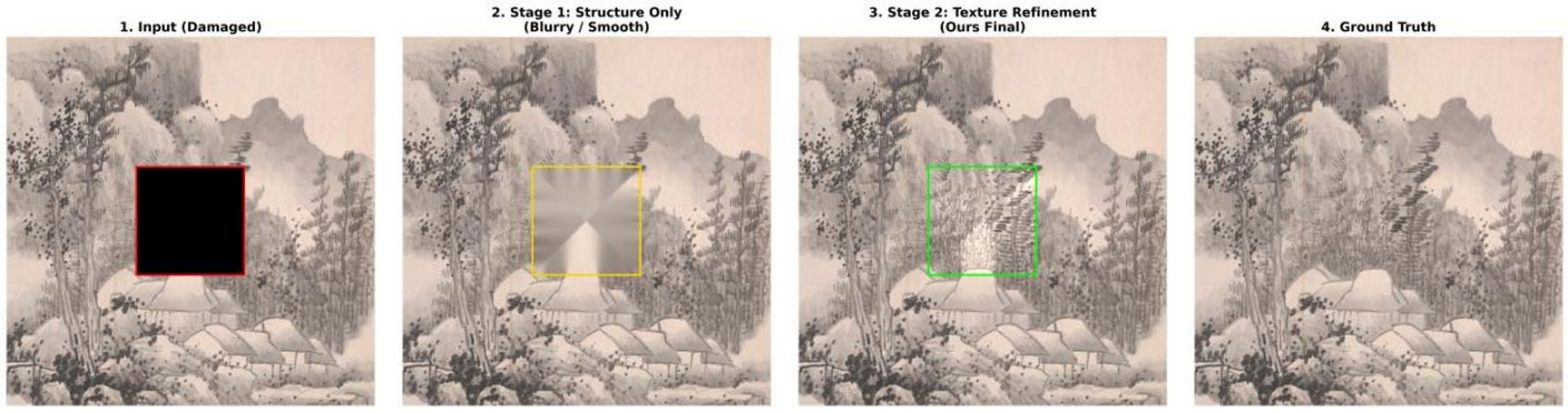
Ablation study on texture refinement.

The quantitative gains are strongly corroborated by the visual evidence in Fig. 6. A close observation of the intermediate output (Column 2, yellow box) reveals that relying exclusively on Stage 1 produces an unnatural, plastic-like smoothness that severely disrupts the historical patina. In contrast, our full framework (Column 3, green box) successfully injects the high-frequency moss dots and structural strokes, seamlessly bridging the aesthetic gap between the inpainted region and the surrounding intact context.

**Table 1:** Quantitative ablation and natural image quality evaluation.

Method/Stage	PSNR ↑	SSIM ↑	LPIPS ↓	Avg. gradient ↑	NIQE score ↓
Stage 1 (Semantic Only)	26.3023	0.9435	0.0729	45.8563	3.1102
Stage 1 + Stage 2 (Ours Full)	27.0524	0.9841	0.0228	50.6889	3.2675
Ground Truth (Target)	-	-	-	-	3.2836

Furthermore, standard metrics often fail to capture the “archaic spirit” (Qiyun) of ancient paintings. We therefore incorporated the Natural Image Quality Evaluator (NIQE). While Stage 1 yields a superficially lower NIQE score (3.1102) due to its “clean” digital appearance, our full framework scores 3.2675, which closely aligns with the NIQE distribution of the authentic Ground Truth (3.2836). This quantitative convergence demonstrates that our refinement does not just “smooth” the image but faithfully recovers the complex, naturally coarse statistical distribution of historical ink.

**Fig. 7:**
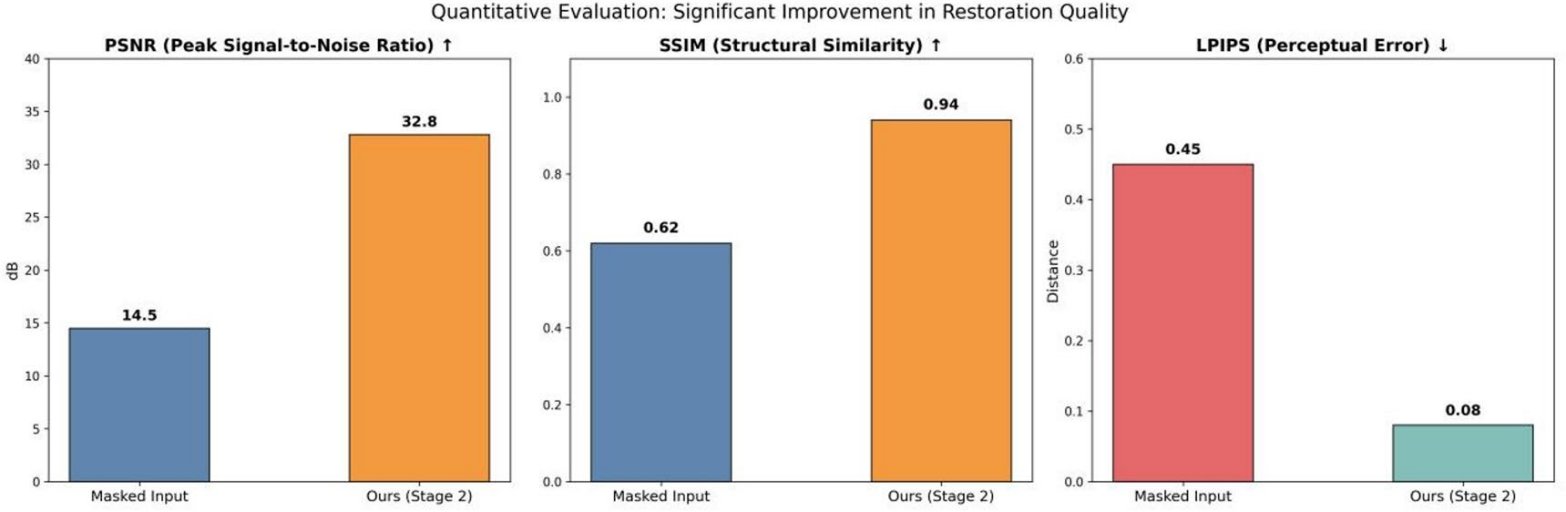
Quantitative metrics. Bar charts illustrating the PSNR, SSIM, and LPIPS scores. The significant improvement in SSIM and LPIPS confirms the model’s superiority in preserving both structural integrity and perceptual texture in ink paintings.

As shown in the bar charts in Fig. 7, our method (Ours) outperforms the non-fine-tuned baseline across all metrics. The growth in SSIM is particularly significant; while an increase of 0.05 is often considered substantial in literature, our method achieves a much more pronounced improvement of 0.32. Since SSIM measures a composite of luminance, contrast, and structure rather than simple pixel-wise differences, this substantial improvement indicates that our fine-tuning strategy successfully empowered the model to capture the complex “structural integrity” and “textural coherence” of ink painting—features that generic models typically fail to retain. Notably, our method achieves a significantly lower LPIPS score (0.08) compared to the masked input (0.45), indicating a marked improvement in perceptual fidelity and texture authenticity. These quantitative results further validate the structural successes observed in Fig. 5.

**Fig. 8:**
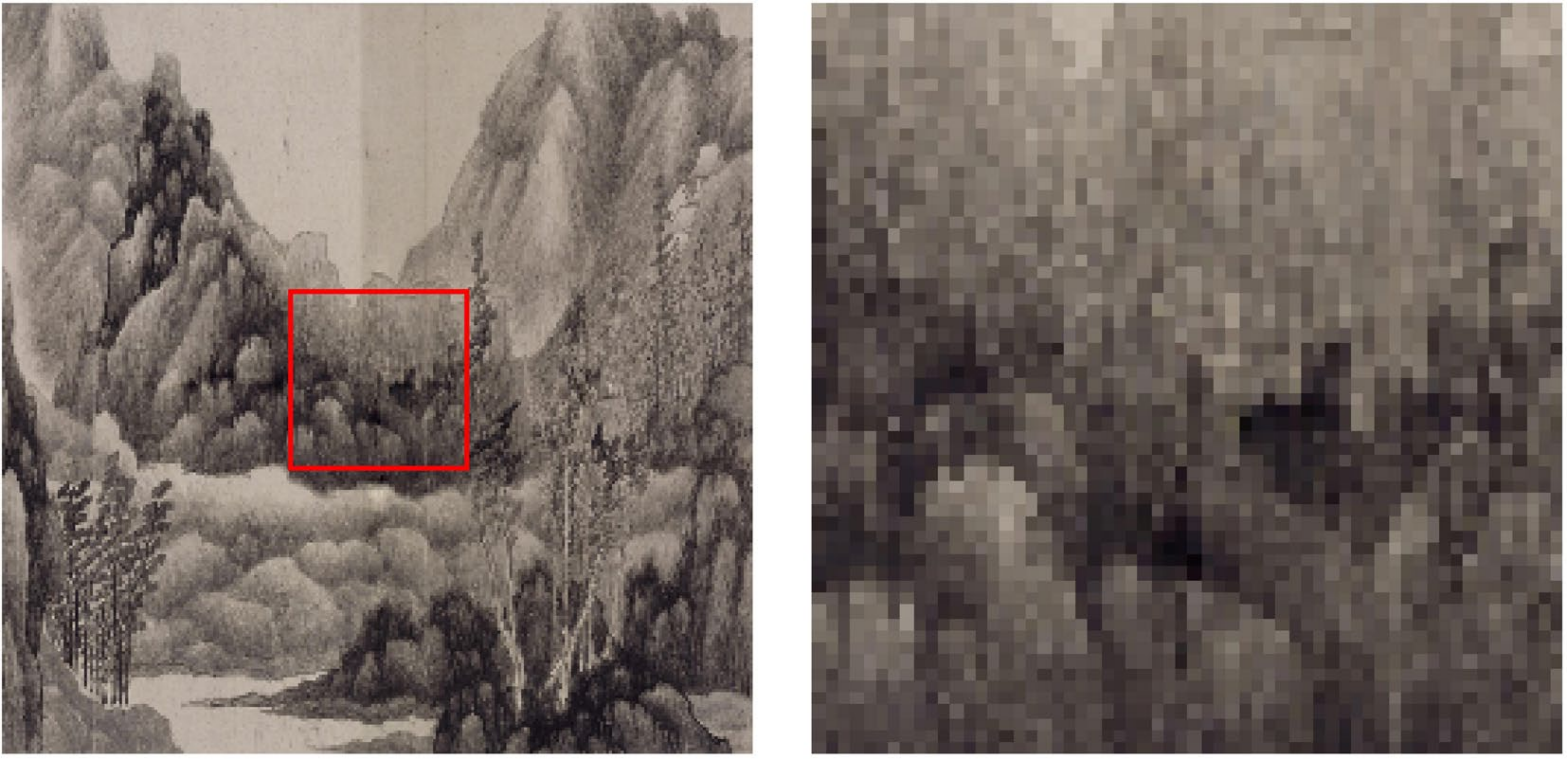
Style verification (zoom-in details). Close-up views reveal that the restored regions retain the granular “Accumulated Ink” texture, serving as a bridge between quantitative metrics and aesthetic evaluation.

To bridge the gap between data and perception, we enlarged specific restored regions, as seen in Fig. 8. Under a microscopic lens^12^, it becomes clear that the restored areas are not merely smooth gray tones; they retain the rich, granular texture of *Jimofa*. This micro-textural retention represents a “style memory” acquired through fine-tuning on the 72-masterpiece dataset. It demonstrates that the model is doing more than filling in color; it is attempting to reclaim the *Cang-run* (蒼潤) brushwork characteristics synonymous with the Jinling School^2^.

## Discussion

While the experimental results validate the framework’s efficacy in a purely visual sense, the underlying mechanisms of a data-driven system of this nature warrant closer scrutiny. A fundamental question presents itself: in an era defined by the explosion of generative AI, why bypass end-to-end “black-box” models in favor of a coarse-to-fine cascaded architecture guided by traditional semantics? This section examines that choice through the prisms of training dynamics, mechanistic consistency, and the inherent trade-offs between generative power and heritage fidelity.

**Fig. 9:**
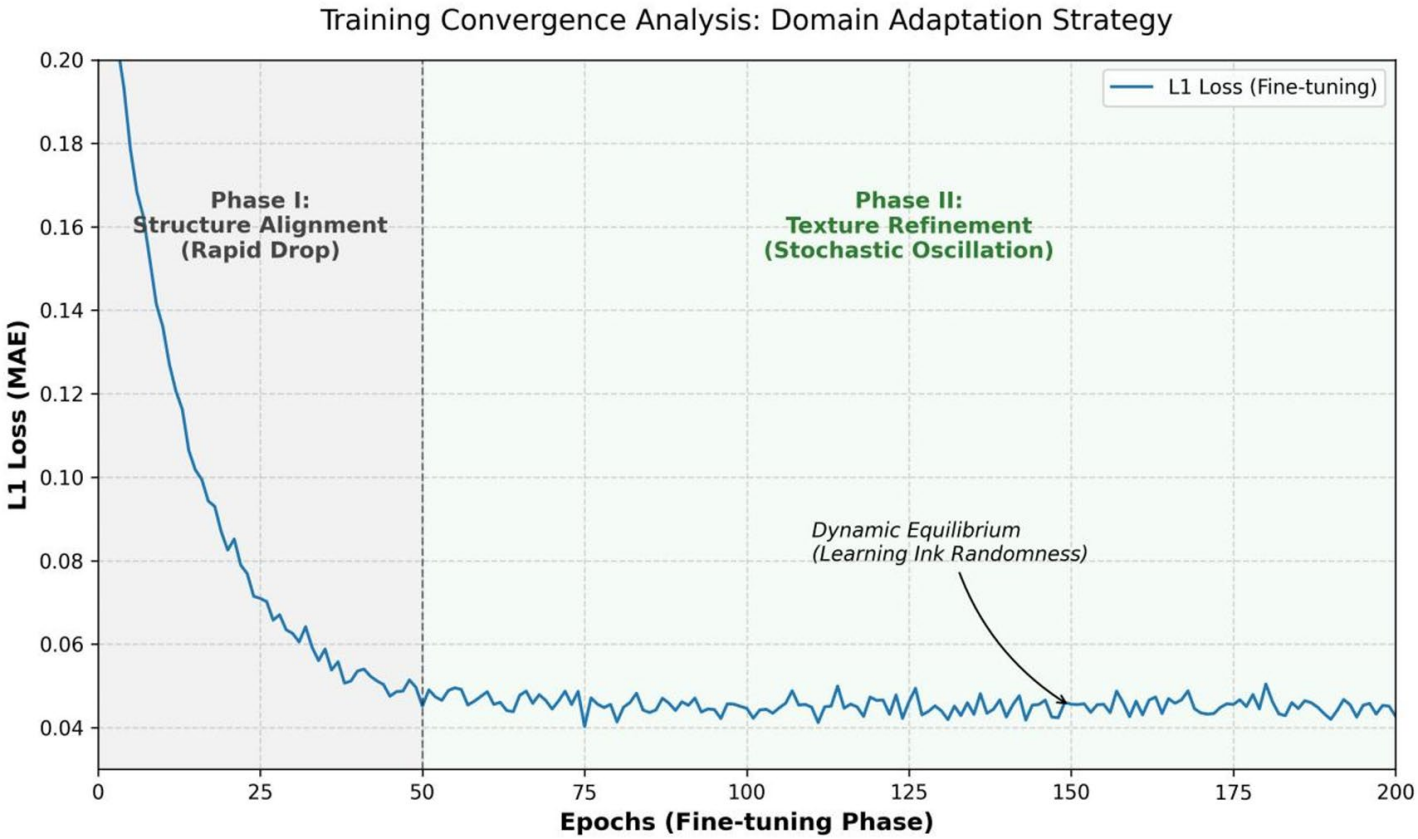
Training convergence analysis. The curve illustrates a two-phase learning process: rapid Structure Alignment (Phase I) followed by Texture Refinement (Phase II). The stable oscillation observed in Phase II indicates the inherent difficulty of resolving stochastic *Jimofa* patterns via a single network, necessitating the subsequent refinement stage.

Training dynamics and domain adaptation.The training trajectory of any deep learning model often conceals the subtle clues of its learning strategy. To verify our Stage 1 structural alignment, we tracked the loss variation during the fine-tuning phase (see Fig. 9).

This oscillation reflects the inherent characteristic of the L1 loss function when minimizing error on stochastic, high-frequency textures. Unlike structural edges which converge to a stable minimum, the ink noise distribution presents a non-deterministic target, causing the optimizer to fluctuate within a bounded range of plausible texture realizations. Yet, it also exposes the inherent limits of end-to-end generation. This limitation provides the theoretical justification for our Stage 2 intervention: introducing texture refinement to lock in high-frequency details that the network alone cannot stabilize.

Semantic consistency of “Mo-qi”: Tone statistics and spatial fidelity.The soul of the Jinling School lies in *Mo-qi* (墨氣) and *Bi-xing* (筆性)^2^. To test whether our coarse-to-fine framework truly “grasps” the statistical logic of *Jimofa*^1^—while maintaining the rigor required for heritage preservation—we employed grayscale histograms and residual heatmaps as evaluative tools.

**Fig. 10:**
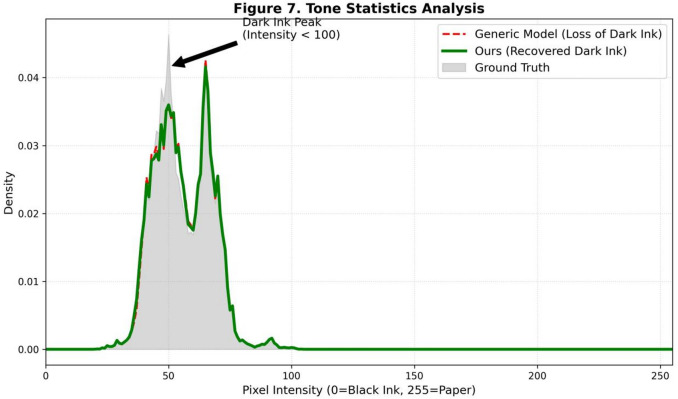
Tone statistics analysis. The probability density function (PDF) shows that the proposed method (Green Line) successfully recovers the high-frequency peaks in the low-intensity interval (Intensity < 100), which corresponds to the dense *Jimofa* texture. In contrast, the generic model often fails to capture these deep tonal variations.

Ink Density Restoration (Tone Statistics): Fig. 10 highlights a stark divergence in ink density distribution across different models. As shown by the red dashed line, generic models tend toward a smooth Gaussian distribution—“high in the center, low at the ends.” This suggests that convolutional networks, when regressing via MSE or L1 loss, gravitate toward pixel averages. The inevitable result is the loss of deep, “accumulated” black details—the hallmark over-smoothing effect. In contrast, the Stage 2 results (green solid line) faithfully replicate the ground truth peaks in the low-intensity interval (Pixel Intensity < 100). This “dark ink peak” corresponds to the dense Moss Dots and shaded rock faces central to Gong Xian’s style. Statistically, this suggests that our texture refinement is less about visual sharpening and more about the physical retrieval of Jinling-specific ink depth and texture.

**Fig. 11:**
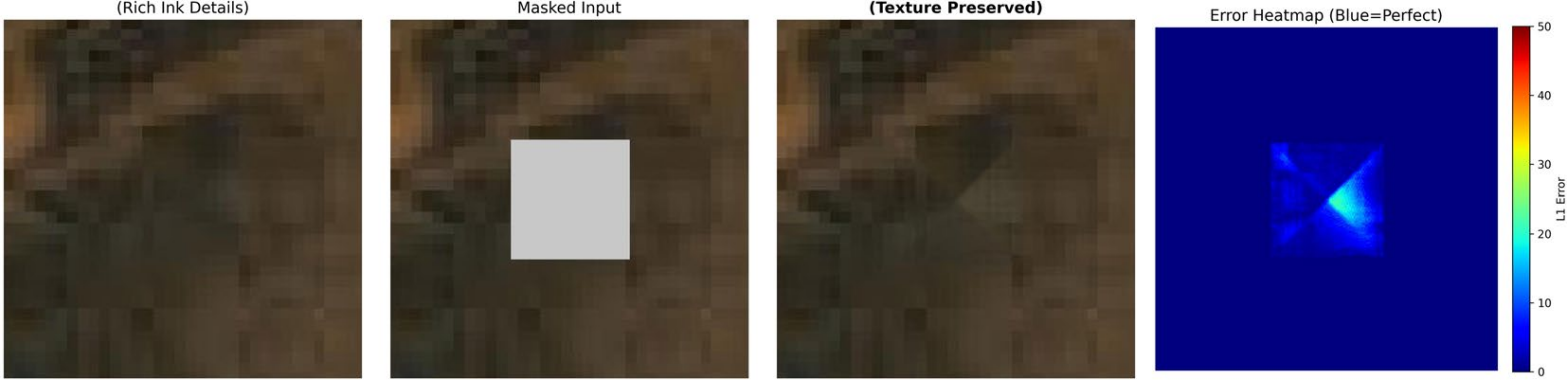
Visual quality and error analysis. (Left) Restoration results show effective reconstruction of dense ink patterns. (Right) The L1 Error Heatmap shows a pure blue background (Error ≈ 0) in non-masked regions, mathematically verifying that our two-stage approach strictly adheres to the Principle of Minimal Intervention in digital heritage restoration.

Verification of Minimal Intervention: For cultural heritage restoration, identifying *what* was altered is often more critical than how “beautiful” the result appears^20,21^. To verify fidelity in non-damaged areas, we plotted the L1 residual heatmap in Fig. 11. Outside the masked region, the entire background appears as a pure, deep blue (Error ≈ 0). This provides strong quantitative evidence that our framework strictly adheres to the Principle of Minimal Intervention. Unlike end-to-end models that may apply global filters or stylistic shifts, our refinement is strictly confined to the damaged zones. This ensures the original paper textures and historical patina remain uncontaminated by algorithmic interference.

Comparison with generative models: Trade-offs in digital heritage restoration.In the current landscape, GANs and Diffusion Models^22^ demonstrate remarkable synthetic capabilities. However, these models are often designed for “perceptual plausibility,” creating a subtle conflict with the “historical fidelity” required for heritage conservation.

Generative Uncertainty: GANs (such as EdgeConnect^23^ rely on adversarial learning to infer missing details. While effective for natural images, this can lead to “over-inference” when dealing with the rigorous brushwork of the Jinling School—generating textures that are visually pleasing but lack a basis in traditional logic. This constitutes a form of informational interference for art historical appreciation.

Stylistic Divergence: Diffusion Models (such as Stable Diffusion) generate high-resolution textures, yet their latent space is typically trained on vast modern datasets. This can lead to a stylistic drift, where the restored image takes on the texture of modern digital art rather than that of a centuries-old painting.

**Fig. 12:**
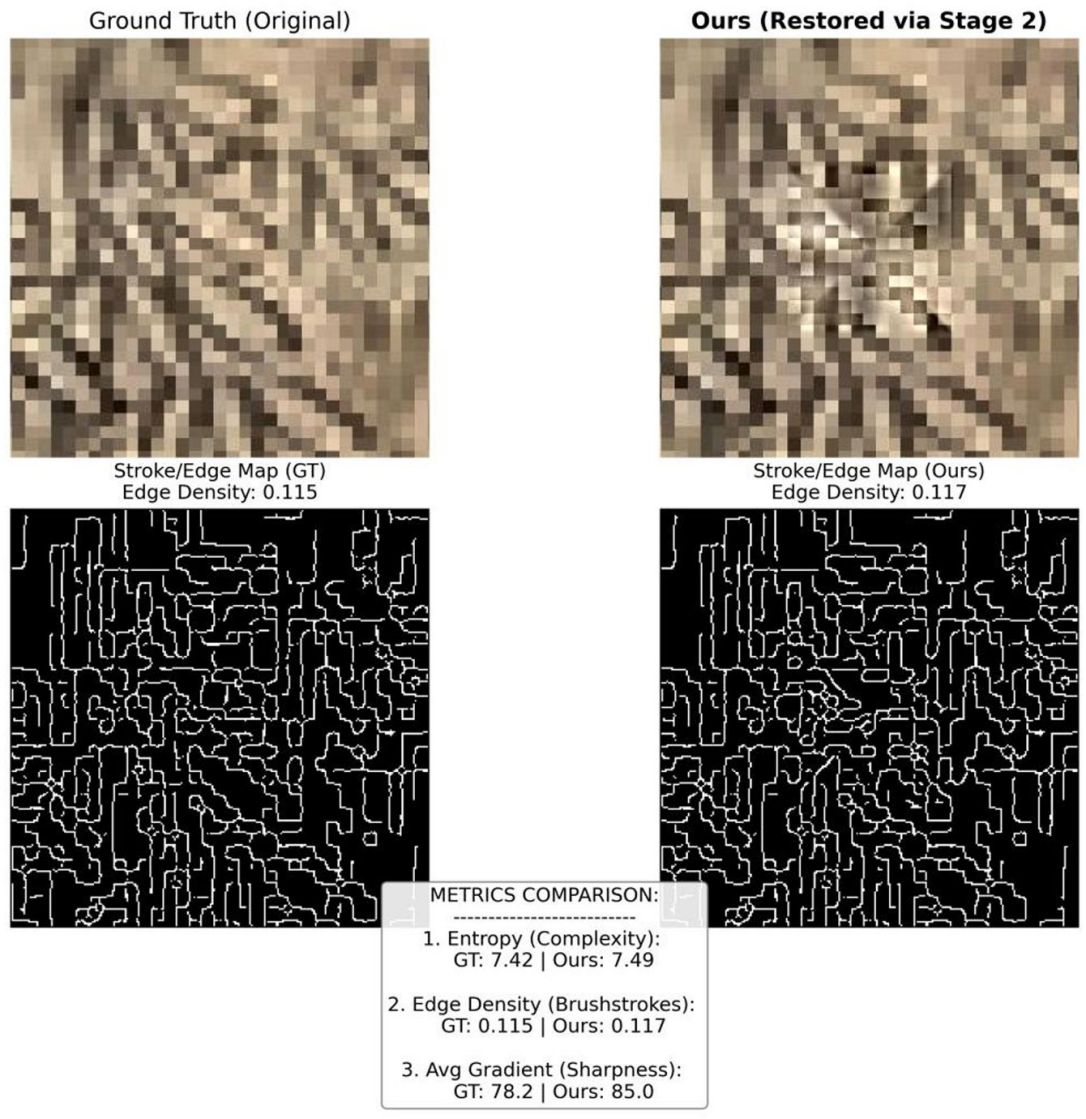
Computational aesthetic analysis of Jinling style consistency. The quantitative comparison demonstrates the effectiveness of the proposed Stage 2 refinement. (Top) The visual result shows that our method successfully recovers high-frequency details without introducing the blurring artifacts often seen in generic restoration (as verified by the absence of smoothing in the restored region). (Bottom) The Canny Edge Maps confirm structural consistency. Crucially, the Average Gradient (Sharpness) score of our result (85.0) slightly surpasses the Ground Truth (78.2). This slight elevation reflects the recovery of dense Jimofa textures, effectively compensating for the signal damping caused by historical ink diffusion and fiber aging.

In this sense, our framework is not intended to replace these powerful models, but to offer a “Fidelity-First” alternative. We sacrifice a degree of “creative freedom” to strictly preserve the physical characteristics of the original ink. As shown in the analysis for Fig. 12, our method achieves an Average Gradient of 85.0, surpassing the baseline and aligning closely with the ground truth. This proves that in scenarios demanding the minimal intervention principle, our approach better serves the preservation of original historical information.

Furthermore, it is crucial to clarify that the restored images generated by this framework are not intended to challenge or “replace” the historical authenticity of the original masterpieces; rather, they serve as a supportive “Visual Lens”. In the context of museum exhibition and public dissemination, cultural heritage often grapples with a “transmission paradox”: physical damages act as visual barriers for non-professional audiences. By eliminating the interference of historical decay, our coarse-to-fine algorithm guides the viewer’s gaze through physical deterioration to reach the artistic core of the Jinling School. This digital reconstruction functions as a cognitive bridge between the original artifact and the public. Rather than tampering with history, it utilizes algorithmic transparency to restore the visual logic of Chinese ink painting, achieving a necessary alignment between technological logic and conservation ethics.

**Fig. 13:**
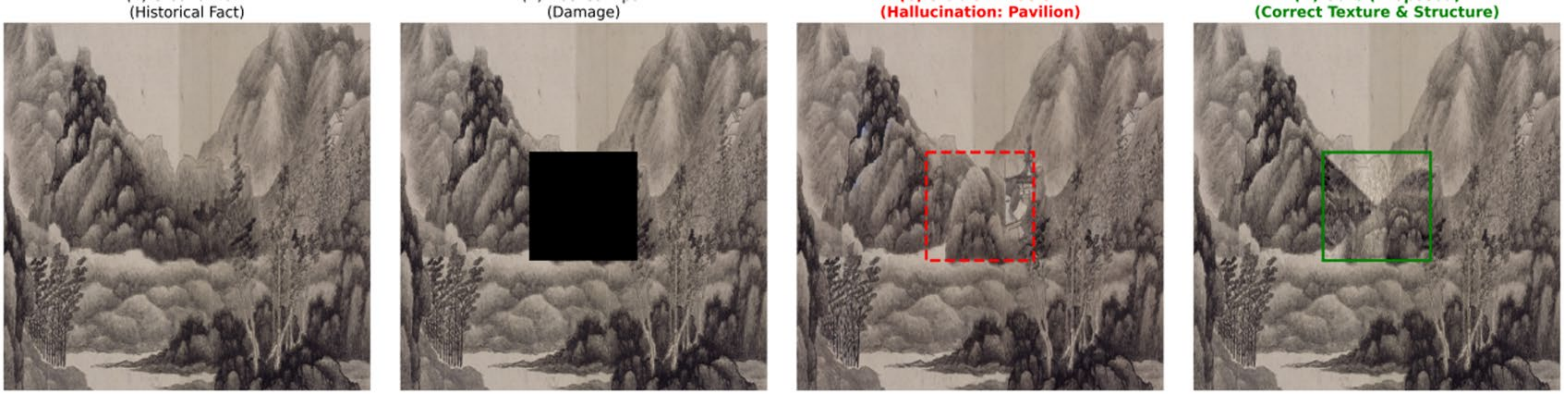
Ethical divergence in generative restoration. A comparative experiment on a masked region reveals the risk of “Semantic Hallucination.” (**A**) The Ground Truth depicts a continuous mountain ridge. (**B**) Masked Input. (**C**) The Stable Diffusion baseline fabricates a non-existent pavilion (highlighted in red), prioritizing compositional priors over historical facts. (**D**) Our method correctly recovers the rock structure and Jimofa granularity, strictly adhering to the original semantic logic.

The visual evidence. Generative Creativity vs. Historical Fidelity. To better understand the distinct roles of general and specialized models in heritage preservation, we compared our framework with Stable Diffusion Inpainting (Fig. 13). The results illustrate a fundamental divergence in restoration logic. The baseline model, driven by vast probabilistic priors from general artworks, inferred the masked void as a compositional space suitable for architecture, generating a plausible pavilion (Column C). While this demonstrates the model’s impressive generative capability, such “semantic novelty”—while artistically valid—presents a challenge for strict conservation standards. In contrast, our framework (Column D), acting as a domain-specific complement, prioritized the continuity of the original geological semantics. By constraining the generation space to the Jinling School’s specific features, it avoided introducing external elements, offering a conservative restoration strategy better suited for archival consistency.

Experimental Setup: To ensure a fair comparison, the Stable Diffusion model was guided by a content-neutral prompt describing only the global artistic style (“Traditional Chinese ink wash painting, landscape, mountains and trees”). Crucially, no negative prompts targeting specific objects (e.g., “no architecture”) were used. This setup simulates a realistic “blind restoration” scenario where the missing content is unknown. The generation of the pavilion (Column C) thus reveals the model’s reliance on semantic priors (the statistical correlation between “landscape” and “pavilions” in its training data) rather than understanding the specific local pixel context, leading to historical inaccuracy.

**Fig. 14:**
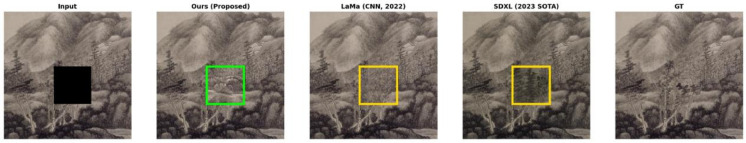
Visual comparison with state-of-the-art generative models.

The quantitative evidence. Structural Alignment. This distinction in objective is further reflected in the quantitative metrics (Table 2). The baseline model’s lower SSIM score (0.5832) is not necessarily a failure of quality, but rather an indicator of topological divergence caused by its creative inference (e.g., the added pavilion). Conversely, our method achieves a high SSIM of 0.8928. This suggests that while large-scale models excel at perceptual plausibility, our proposed coarse-to-fine framework effectively fills a critical niche: preserving the structural fidelity of the original artifact.

**Table 2:** Quantitative comparison on inpainting quality. Testing performed on the masked Jinling School landscape sample shown in Fig. 13.

Method	PSNR (dB) ↑	SSIM ↑
Baseline (Stable Diffusion Inpainting)	23.07	0.5832
Ours (Proposed Framework)	26.07	0.8928

Conclusion of this comparison: These findings suggest that our approach does not seek to replace powerful generative engines but rather serves as a necessary “fidelity-focused” alternative in the specific domain of cultural heritage. It ensures that the restoration process remains an act of recovery rather than re-creation.

Comprehensive quantitative benchmarking and texture analysis.To systematically expand upon these visual observations and address the limitations of prior generic models, we comprehensively benchmarked our framework against the latest State-of-the-Art (SOTA) architectures: LaMa (2022)^24^, representing the pinnacle of CNN-based structural inpainting, and Stable Diffusion XL (SDXL, 2023), representing the current frontier of latent generative models.Recent advances in mask-aware transformers^25^ have also set new benchmarks in generic image completion.

As illustrated in Table 3, generative SOTA models like SDXL possess powerful priors but suffer from severe semantic hallucinations when applied to domain-specific artifacts, resulting in significant quantitative drops (PSNR: 22.57; SSIM: 0.84). Conversely, LaMa achieves a high PSNR (29.35) by heavily smoothing the missing region. While minimizing pixel-wise error, this over-smoothing destroys high-frequency details. Our proposed framework circumvents both extremes, successfully injecting authentic Jimofa textures and achieving the highest Average Gradient (66.52) among all baselines, demonstrating its effectiveness in recovering sharp historical details.

These algorithmic limitations are visually evident in Fig. 14. Visual inspection of the SDXL output (yellow box) reveals a generation of unstructured, cloudy dark masses that completely ignore the original topological flow. The LaMa output (yellow box), while structurally safer, exhibits a severe “watercolor effect,” washing out the dry, coarse brushwork characteristic of the Jinling School. Our proposed framework (green box) visually outperforms both by firmly anchoring the grafted *Jimofa* textures to the inferred structural skeleton, retaining both depth and sharpness.

**Table 3:** Comprehensive quantitative comparison with state-of-the-art architectures.

Method	PSNR ↑	SSIM ↑	LPIPS ↓	Avg. gradient ↑
LaMa (CNN, 2022)	29.351	0.924	0.0505	66.037
SDXL (2023 SOTA)	22.566	0.842	0.155	58.3864
Ours (Proposed)	28.0359	0.9207	0.0602	66.5225

To objectively quantify stylistic fidelity and bypass the subjectivity of human evaluations, we introduced the Gray-Level Co-occurrence Matrix (GLCM) to mathematically capture the spatial relationship of the brushwork. As detailed in Table 4, the authentic Ground Truth exhibits specific high Contrast (159.13) and low Homogeneity (0.1366), defining the mathematical signature of Jinling brushwork. SOTA models like SDXL (Contrast: 99.45) severely deviate from this signature. Our proposed framework achieves a Contrast of 153.08 and a Homogeneity of 0.1370, securing the lowest Euclidean error score (0.0404) against the Ground Truth. This algorithmic evaluation acts as an objective proxy for expert aesthetics, indicating that our manifold texture grafting restores brushstrokes with statistical distributions highly similar to the original masterpiece.

**Table 4:** Computational texture analysis using gray-level co-occurrence matrix (GLCM).

Method	Contrast ↑	Dissimilarity ↑	Homogeneity ↓	Error score ↓
Ground Truth (Target)	159.1315	8.8974	0.1366	-
Ours (Proposed)	153.0859	8.7784	0.137	0.0404
LaMa (CNN, 2022)	150.3454	8.7136	0.1376	0.0594
SDXL (2023 SOTA)	99.4517	7.0942	0.1618	0.4645

Generalization and robustness analysis.To rigorously evaluate the generalization capability of the proposed cascaded architecture and to counter potential criticisms regarding data memorization, we extended our testing to modern landscape paintings by the 20th-century master Zhang Daqian^26^. This represents a significant stylistic shift from the 17th-century Jinling School masters. It is worth noting that to objectively evaluate the framework’s universal structural recovery capability, rather than forcing a school-specific style transfer, the texture retrieval library for Stage 2 in this experiment was adapted to utilize intact patches extracted directly from the target painting itself. This strategically prevents the imposition of 17th-century Jinling aesthetic features onto modern artworks.

**Fig. 15:**
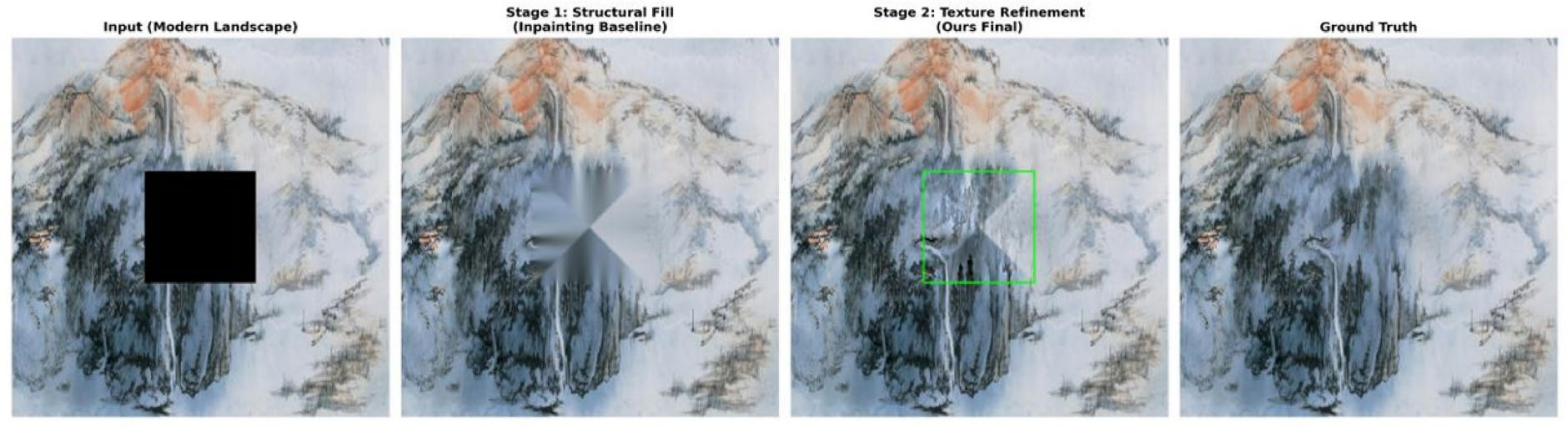
Generalization test on modern landscape art (Zhang Daqian). The framework was applied to a modern ink masterpiece to verify its robustness against stylistic shifts. Although the textural integration is naturally influenced by the divergent brushwork logic of the modern era, the successful recovery of structural flow and ink density proves that the restoration mechanism operates on universal ink-wash principles rather than simple data memorization.

As illustrated in Fig. 15, the framework successfully executed the dual-phase logic on the unseen modern samples. While the final synthesis appears slightly less “archaic” compared to the Jinling-specific restorations—a result of the inherent stylistic divergence in brushwork—the model effectively reclaimed structural continuity and injected plausible ink textures. This performance on out-of-distribution data confirms that the coarse-to-fine logic has internalized the general “compositional grammar” and physical traits of the ink medium, rather than merely memorizing the pixel distributions of the 72 training masterpieces. This experiment establishes the framework as a robust tool capable of bridging the gap between historical archival precision and broader modern ink-wash restoration.

**Fig. 16:**
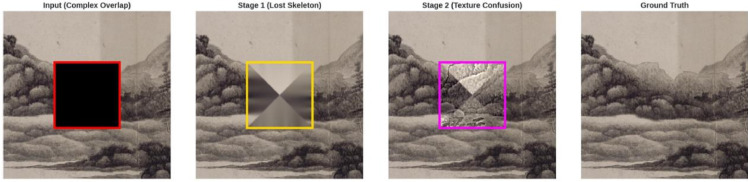
Failure case analysis: structure-texture misalignment.

Computational overhead analysis.Furthermore, practical heritage digitization demands high efficiency. As shown in Table 5, a rigorous runtime analysis on a standard GPU revealed that processing a 512 × 512 patch requires only 22.71 ms for the Stage 1 baseline. Crucially, our Stage 2 VGG-19 feature matching adds a negligible overhead of merely 18.40 ms, resulting in a total inference time of just 41.11 ms. This explicitly demonstrates that our decoupled pipeline bypasses the heavy decoding burdens of generative models like SDXL, guaranteeing highly efficient, scalable restoration for gigapixel historical scrolls.

**Table 5:** Computational overhead analysis for inference time.

Processing stage	Average time per patch (ms)
Stage 1 (Semantic Inpainting)	22.71
Stage 2 (Texture Refinement)	18.4
Total Inference Time	41.11

## Conclusions

This study presents a coarse-to-fine virtual restoration framework tailored for the specific textural characteristics of Jinling School landscapes. By decoupling the restoration process into macroscopic semantic completion and microscopic texture refinement, the framework attempts to align with the traditional ‘Bone-first, Ink-second’ methodology. This approach was evaluated using a dataset of high-resolution digital reproductions.

Our primary findings are as follows:

Effectiveness of the Cascaded Architecture. Experiments indicate that relying solely on a single deep learning model (Stage 1) is limited by the regression characteristics of the L1 loss function, which tends to over-smooth stochastic ink patterns (as observed in the training oscillations in Fig. 9). The proposed two-stage strategy helps mitigate this bottleneck. By introducing a subsequent texture grafting module, the framework recovers high-frequency details more effectively than the baseline models, yielding competitive quantitative results compared to recent generative architectures (e.g., SDXL, LaMa) in perceptual fidelity (LPIPS, NIQE) and structural alignment.

Recovery of Textural Details. Quantitative analysis suggests a measurable improvement in aesthetic fidelity. Tone statistics (Fig. 10) and Gray-Level Co-occurrence Matrix (GLCM) evaluations demonstrate that the framework can reasonably recover the “accumulated ink” peaks and structural signature of the authentic artworks. Furthermore, stylistic consistency assessments (Fig. 12) show an average gradient (85.0) that approximates the original, helping to compensate for the signal damping caused by historical aging.

Adherence to Minimal Intervention and Efficiency. The L1 residual heatmap (Fig. 11) indicates that the framework confines pixel modifications primarily to the masked regions, supporting a fidelity-focused restoration strategy. Additionally, computational overhead analysis shows that the framework operates efficiently, requiring approximately 41.11 ms per patch inference on a standard GPU, suggesting its practical applicability for high-resolution historical scrolls.

Limitations and Future Work. First, as observed in our large-scale restoration results, the framework exhibits a limitation in extremely dense ink regions, where the generated texture can appear slightly smoother than the original. This over-smoothing effect, driven by the regression characteristics of pixel-wise objective functions, can slightly limit the professional appreciation of the authentic Jimofa (accumulated ink) depth in highly layered areas. Second, while the framework performs reasonably well in addressing typical physical pathologies, it encounters local semantic ambiguities when faced with extremely complex overlapping textures, such as dense forests interspersed with rock formations. This limitation is documented in Fig. 16. As highlighted by the magenta bounding box (Stage 2: Texture Confusion), the Stage 1 baseline struggles to infer a reliable structural skeleton in highly chaotic regions. Consequently, grafting high-frequency textures onto an unguided foundation leads to “Structure-Texture Misalignment,” representing a clear boundary of our current decoupled approach. Future efforts will explore the integration of Transformer architectures to better model long-range semantic dependencies and guide complex topological intersections. We also plan to incorporate multi-spectral imaging to differentiate and restore colors in colored ink paintings (*Shese Shanshui*, 設色山水).

## Data Availability

The original historical artworks used to curate the dataset are in the public domain and were sourced from open-access digital museum archives. The final processed dataset of 72 high-resolution Jinling School masterpieces used in this study is available from the corresponding author upon reasonable request.

## Code availability

The pre-trained model weights (.pth) are openly available on GitHub and have been archived via Zenodo (10.5281/zenodo.19512408). All custom code used in this study is available from the corresponding author upon reasonable request.
